# Coronary Artery Disease: Optimal Lipoprotein(a) for Survival—Lower Is Better? A Large Cohort With 43,647 Patients

**DOI:** 10.3389/fcvm.2021.670859

**Published:** 2021-08-31

**Authors:** Jin Liu, Liwei Liu, Bo Wang, Shiqun Chen, Buyun Liu, Jingjing Liang, Haozhang Huang, Qiang Li, Zhubin Lun, Ming Ying, Guanzhong Chen, Zhidong Huang, Danyuan Xu, Xiaoming Yan, Tingting Zhu, Girmaw Abebe Tadesse, Ning Tan, Jiyan Chen, Yong Liu

**Affiliations:** ^1^Department of Cardiology, Guangdong Provincial Key Laboratory of Coronary Heart Disease Prevention, Guangdong Cardiovascular Institute, Guangdong Provincial People's Hospital, Guangdong Academy of Medical Sciences, Guangzhou, China; ^2^Department of Epidemiology, College of Public Health, University of Iowa, Iowa City, IA, United States; ^3^The Second School of Clinical Medicine, Southern Medical University, Guangzhou, China; ^4^Department of Cardiology, Dongguan TCM Hospital, Dongguan, China; ^5^Guangdong Provincial People's Hospital, School of Medicine, South China University of Technology, Guangzhou, China; ^6^Department of Information Technology, Guangdong Provincial People's Hospital, Guangdong Academy of Medical Sciences, Guangzhou, China; ^7^Department of Engineering Science, Institute of Biomedical Engineering, University of Oxford, Oxford, United Kingdom

**Keywords:** coronary artery disease, baseline lipoprotein(a), long-term all-cause mortality, percutaneous coronary intervention, coronary angiography

## Abstract

**Background:** A high level of lipoprotein(a) can lead to a high risk of cardiovascular events or mortality. However, the association of moderately elevated lipoprotein(a) levels (≥15 mg/dL) with long-term prognosis among patients with coronary artery disease (CAD) is still uncertain. Hence, we aim to systematically analyzed the relevance of baseline plasma lipoprotein(a) levels to long-term mortality in a large cohort of CAD patients.

**Methods:** We obtained data from 43,647 patients who were diagnosed with CAD and had follow-up information from January 2007 to December 2018. The patients were divided into two groups (<15 and ≥15 mg/dL). The primary endpoint was long-term all-cause death. Kaplan–Meier curve analysis and Cox proportional hazards models were used to investigate the association between moderately elevated baseline lipoprotein(a) levels (≥15 mg/dL) and long-term all-cause mortality.

**Results:** During a median follow-up of 5.04 years, 3,941 (18.1%) patients died. We observed a linear association between lipoprotein(a) levels and long-term all-cause mortality. Compared with lipoprotein(a) concentrations <15 mg/dL, lipoprotein(a) ≥15 mg/dL was associated with a significantly higher risk of all-cause mortality [adjusted hazard ratio (aHR) 1.10, 95%CI: 1.04–1.16, *P*-values = 0.001). Similar results were found for the subgroup analysis of non-acute myocardial infarction, non-percutaneous coronary intervention, chronic heart failure, diabetes mellitus, or non-chronic kidney diseases.

**Conclusion:** Moderately elevated baseline plasma lipoprotein(a) levels (≥15 mg/dL) are significantly associated with higher all-cause mortality in patients with CAD. Our finding provides a rationale for testing the lipoprotein(a)-reducing hypothesis with lower targets (even <15 mg/dL) in CAD outcome trials.

## Introduction

In the field of epidemiology and genetics, numerous studies have found that plasma lipoprotein(a) remains a residual risk factor for atherosclerotic cardiovascular disease (ASCVD) (1–4). Elevated plasma lipoprotein(a) levels are consequently associated with an increased risk of adverse clinical events because of the atherogenic and thrombogenic properties of lipoprotein(a) (4–7).

The 2019 ESC/EAS dyslipidemia guidelines suggested that lipoprotein(a) should be measured in people with high ASCVD risk, and elevated lipoprotein(a) (≥50 mg/dL) would be regarded as a significant risk factor of cardiovascular disease; however, the dose effect of lipoprotein(a) with long-term prognosis among ASCVD patients is uncertain (8). Several studies found that a baseline plasma lipoprotein(a) >30 mg/dL would increase the risk of cardiovascular events or all-cause mortality (7, 9–11). A Vera et al. indicated that baseline lipoprotein(a) levels were not significantly associated with long-term mortality among ACS patients (12). As a whole, the effect of moderately elevated lipoprotein(a) levels (≥15 mg/dL) for long-term mortality among patients with CAD was still uncertain. The definitive lipoprotein(a) levels may provide a more accurate risk stratification for indicating a high risk of CAD patients and may prompt an important reference for the subsequent improvement of long-term prognosis among CAD patients via a lipoprotein(a)-lowering treatment.

Therefore, we systematically analyzed the relevance of moderately elevated lipoprotein(a) levels (≥15 mg/dL) to long-term mortality in a large cohort of CAD patients.

## Methods

### Study Design and Participants

The study was based on a total of 88,938 patients as confirmed by coronary angiography (CAG) from January 2007 to December 2018 at Guangdong Provincial People's Hospital in China (Clinical Trials.gov NCT04407936), and the retrospective observational study included 59,667 patients with a final diagnosis of CAD according to the 10th Revision Codes of the International Classification of Diseases (ICD-10; I20.xx–I25.xx, I50.00001 and I91.40001, Supplementary Table 2). After excluding 8,723 patients who lacked a lipoprotein(a) examination, a total of 50,944 patients were enrolled in the analyses subsequently, with lack of follow-up information of CAD mortality (*n* = 7,297). The final analysis included 43,647 CAD patients who underwent the first CAG during the study period. The study protocol was approved by the Guangdong Provincial People's Hospital Ethics Committee, and the study was performed according to the Declaration of Helsinki.

Baseline data were extracted from the electronic Clinical Management System of the Guangdong Provincial People's Hospital. The baseline information mainly included demographic characteristics, coexisting conditions, laboratory examinations, and medications at discharge. CAG or percutaneous coronary intervention (PCI) was performed following standard clinical practice guidelines (13–15).

### Clinical Definition

Chronic kidney disease (CKD) was defined as an estimated glomerular filtration rate (eGFR) <60 mL/min/1.73 m^2^, and eGFR was calculated using the Modification of Diet in Renal Disease equation (16). Congestive heart failure (CHF) was defined as New York Heart Association class >2 or Killip class >1. Diabetes mellitus (DM) and hypertension were defined using ICD-10 codes (Supplementary Table 2).

### Study Outcome

The primary endpoint was long-term all-cause death, and incident events were defined as the first event occurring between the date of enrollment and the end of follow-up on December 31, 2018. This information was monitored and recorded by research assistants and trained nurses through outpatient interviews and telephones. The concentration of lipoprotein(a) was measured for the first time in the hospital *via* immunoturbidimetry on a chemistry analyzer (AU5800 Analyzer, Beckman Coulter, Brea, California).

### Statistical Analysis

The statistical analysis for this study was performed from January 1, 2007 to December 31, 2018. The patients were divided into normal group (<15 mg/dL) and moderately elevated group (≥15 mg/dL) by lipoprotein(a) concentration. Continuous variables were tested for normality with visual inspection (histograms and normal Q-Q plot) and expressed as mean (standard deviation, SD). The descriptive statistics for continuous variables with abnormal distribution and categorical variables are reported as median (interquartile range, IQR) and numbers (percentages), respectively. Student's *t*-test and Wilcoxon rank-sum test were used to analyze differences across groups as appropriate. Pearson chi-square tests were used to analyze the categorical data.

Prognosis analysis was performed using Kaplan–Meier methods, and survival curves were used for the probability of the remaining outcome-free in the two groups. We used the log-rank test to compare the survival differences among the two groups.

Cox proportional hazard regression models and restricted cubic splines were used to investigate the associations of baseline lipoprotein(a) levels with long-term all-cause mortality. Hazard ratio and 95% confidence interval (CI) were reported. Model 1 was univariate Cox analysis. Model 2 was to adjust age (as a continuous variable) and gender. Model 3 included age, gender, and four lipid continuous variables (total cholesterol, triglyceride, apolipoprotein A, and low-density lipoprotein cholesterol), and model 4 included the variables which were significant at *P* < 0.05 according to univariate Cox proportional hazard regression and those associated with mortality according to clinical experience (including models 2 and 3 variables, history of present illness information, and drug information). Furthermore, we additionally adjusted apolipoprotein B, fibrinogen, and hypersensitive C-reactive protein, respectively, based on model 4. Finally, we defined the results of model 4 as the primary results. When data were missing, we used the last available observation.

We used model 3 to perform a sensitivity analysis in five different subgroups [PCI, acute myocardial infarction (AMI), CKD, DM, and CHF]. All data analyses were performed using R (version 3.6.3; R Core Team, Vienna, Austria). *P*-values <0.05 were considered to represent statistical significance.

## Result

### Clinical Characteristics

From January 2007 to December 2018, a total of 43,647 CAD patients were enrolled in the study. The mean age was 63.1 ± 10.7 years, and 33,198 (76.1%) were male. The patients were divided into two groups according to the median level of lipoprotein(a) concentration: lower lipoprotein(a) group [lipoprotein(a) <15 mg/dL, *n* = 19,945] and higher lipoprotein(a) group [lipoprotein(a) ≥ 15 mg/dL, *n* = 23,702]. Among the subgroup analyses, 31,688 (72.6%) patients underwent PCI treatment, and 9,541 (22.9%) patients were identified as CKD, 12,008 (27.5%) patients were complicated with DM, and 4,160 (9.5%) had CHF (Table 1).

**Table 1:** Baseline characteristics of the patients.

Characteristic*	**Overall (*n* = 43,647)**	**LPa <15 mg/dL (*n* = 19,945)**	**LPa ≥ 15 mg/dL (*n* = 23,702)**	***P*-value**
**Demographic characteristics**				
Age, years, mean (SD)	63.1 (10.7)	62.8 (10.8)	63.3 (10.6)	<0.001
Female, *n* (%)	10,449 (23.9)	4,649 (23.3)	5,800 (24.5)	0.005
**Medical history**				
AMI, *n* (%)	8,584 (19.7)	3,321 (16.7)	5,263 (22.2)	<0.001
CHF, *n* (%)	4,146 (9.5)	1,611 (8.1)	2,535 (10.7)	<0.001
Hypertension, *n* (%)	24,619 (56.5)	11,436 (57.4)	13,183 (55.7)	<0.001
DM, *n* (%)	12,008 (27.5)	5,703 (28.6)	6,305 (26.6)	<0.001
PCI, *n* (%)	31,688 (72.6)	13,843 (69.4)	17,845 (75.3)	<0.001
CKD, *n* (%)	7,651 (17.5)	3,011 (15.1)	4,640 (19.6)	<0.001
**Laboratory tests**				
Lipoprotein(a), mg/dL, mean (SD)	29.61 (33.18)	8.41 (3.50)	47.44 (36.34)	<0.001
WBC, 10^9^/L, mean (SD)	7.98 (2.73)	7.90 (2.74)	8.05 (2.72)	<0.001
HGB, g/L, mean (SD)	133.28 (16.81)	134.83 (16.11)	131.98 (17.27)	<0.001
CHOL, mmol/L, mean (SD)	4.54 (1.21)	4.38 (1.15)	4.68 (1.24)	<0.001
TRIG, mmol/L, mean (SD)	1.66 (1.22)	1.80 (1.47)	1.54 (0.94)	<0.001
APOA, g/L, mean (SD)	1.54 (0.94)	1.11 (0.26)	1.09 (0.26)	<0.001
APOB, g/L, mean (SD)	0.86 (0.24)	0.82 (0.23)	0.89 (0.25)	<0.001
LDLC, mmol/L, mean (SD)	2.81 (0.97)	2.64 (0.91)	2.95 (1.00)	<0.001
HDLC, mmol/L, mean (SD)	1.00 (0.26)	0.99 (0.26)	1.01 (0.26)	<0.001
HbA1c, %, mean (SD)	6.55 (1.41)	6.54 (1.38)	6.56 (1.44)	0.231
URIC, μmol/L, mean (SD)	396.45 (112.37)	397.36 (109.72)	395.68 (114.56)	0.159
eGFR, mL/min/1.73 m^2^, mean (SD)	77.51 (24.80)	79.49 (24.12)	75.89 (25.22)	<0.001
**Medications**				
ACEI/ARB, *n* (%)	21,180 (49.3)	9,446 (48.1)	11,734 (50.4)	<0.001
Beta-blockers, *n* (%)	34,774 (81.0)	15,760 (80.3)	19,014 (81.6)	<0.001
Statins, *n* (%)	40,715 (94.9)	18,565 (94.6)	22,150 (95.1)	0.012

*LPa, lipoprotein(a); AMI, acute myocardial infarction; CHF, congestive heart failure; DM, diabetes mellitus; PCI, percutaneous coronary intervention; CKD, chronic kidney disease; WBC, white blood cell; HGB, hemoglobin; CHO, serum total cholesterol; TG, triglycerides; APOA, apolipoprotein A; APOB, apolipoprotein B; LDL-C, low-density lipoprotein cholesterol; HDL-C, high-density lipoprotein cholesterol; eGFR, estimated glomerular filtration rate; ACEI/ARB, angiotensin-converting enzyme inhibitor/angiotensin receptor blocker*.

### Primary Outcomes

The median level of lipoprotein was 15.0 mg/dL. In total, there were 3,941 (18.1%) deaths during a median follow-up of 5.0 years (IQR, 3.0–7.8; Table 1), and patients with lipoprotein(a) ≥15 mg/dL has a higher mortality rate than those with lipoprotein(a) <15 mg/dL [3,391 (14.3%) vs. 2,381 (11.9%), *P* < 0.001, respectively]. The level of lipoprotein(a) did not significantly differ with age but was higher in females (Supplementary Figure 2).

**Figure 1:**
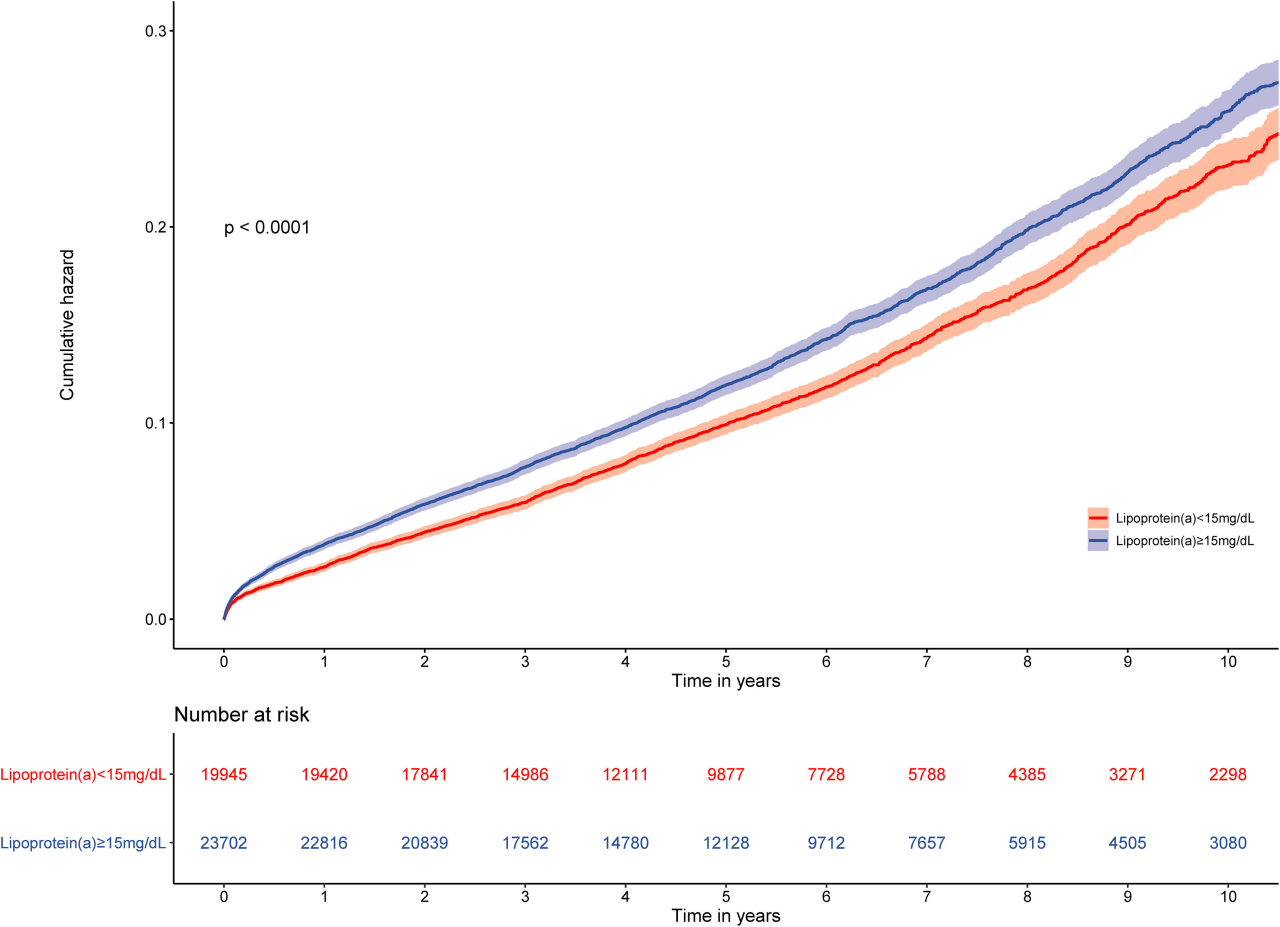
Kaplan–Meier curves for quartile values of plasma levels of lipoprotein(a). Normal baseline lipoprotein(a) levels: <15 mg/dL. Moderately elevated baseline lipoprotein(a) levels: ≥15 mg/dL.

**Figure 2:**
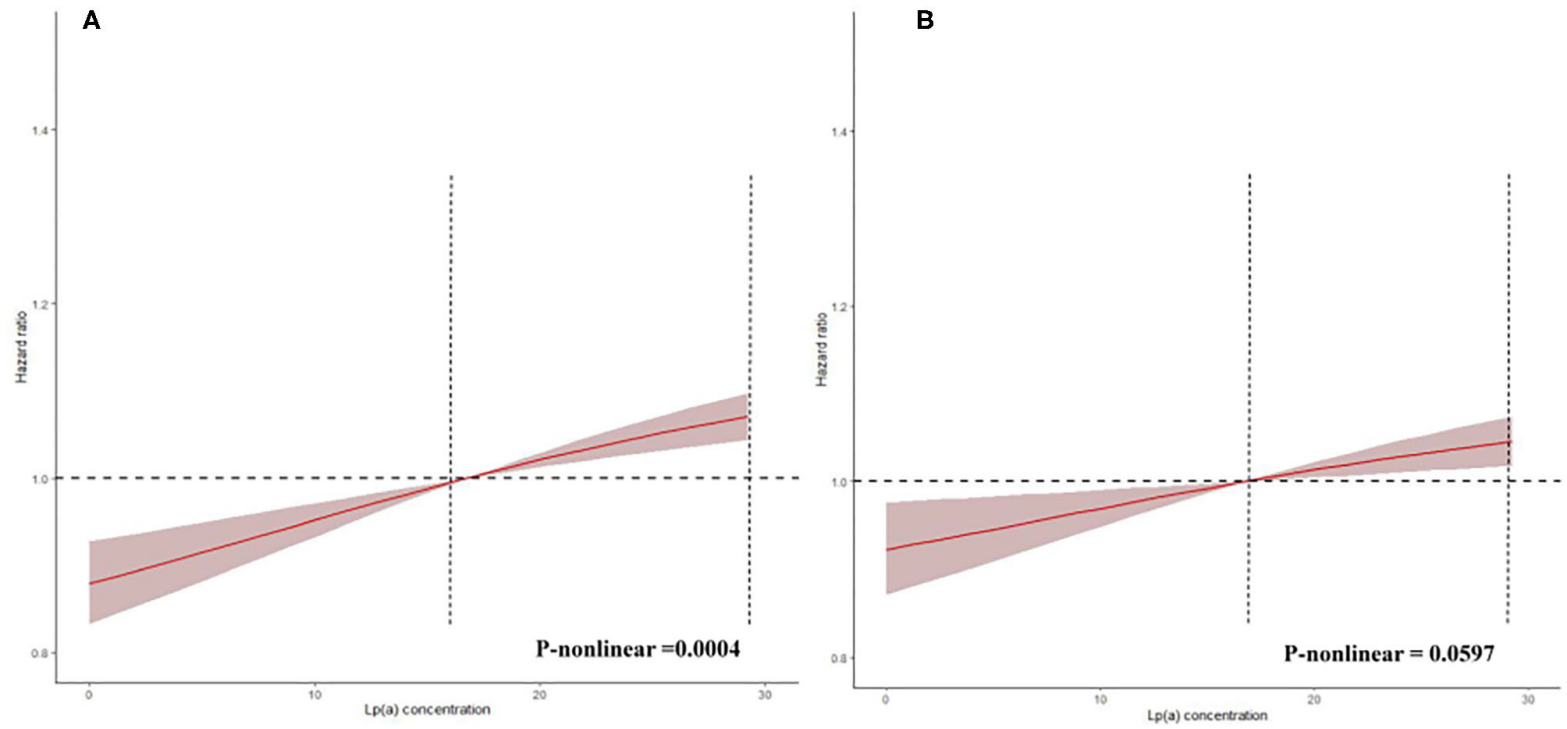
Restricted spline curve for the lipoprotein(a) hazard ratio. This reflects spline of degree 3 (piecewise cubic curve) with natural cubic basis and 3 knots. **(A)** The restrict spline curve of a univariate Cox model. **(B)** The restrict spline curve of a multivariate Cox model, adjusted for age, gender, hypertension, acute myocardial infraction, diabetes mellitus, percutaneous coronary intervention, hemoglobin, white blood cell, congestive heart failure, total cholesterol, triglyceride, apolipoprotein A, low-density lipoprotein cholesterol, high-density lipoprotein cholesterol, chronic kidney disease, angiotensin-converting enzyme inhibitor/angiotensin receptor blockers, β-blockers, and statins.

**Figure 3:**
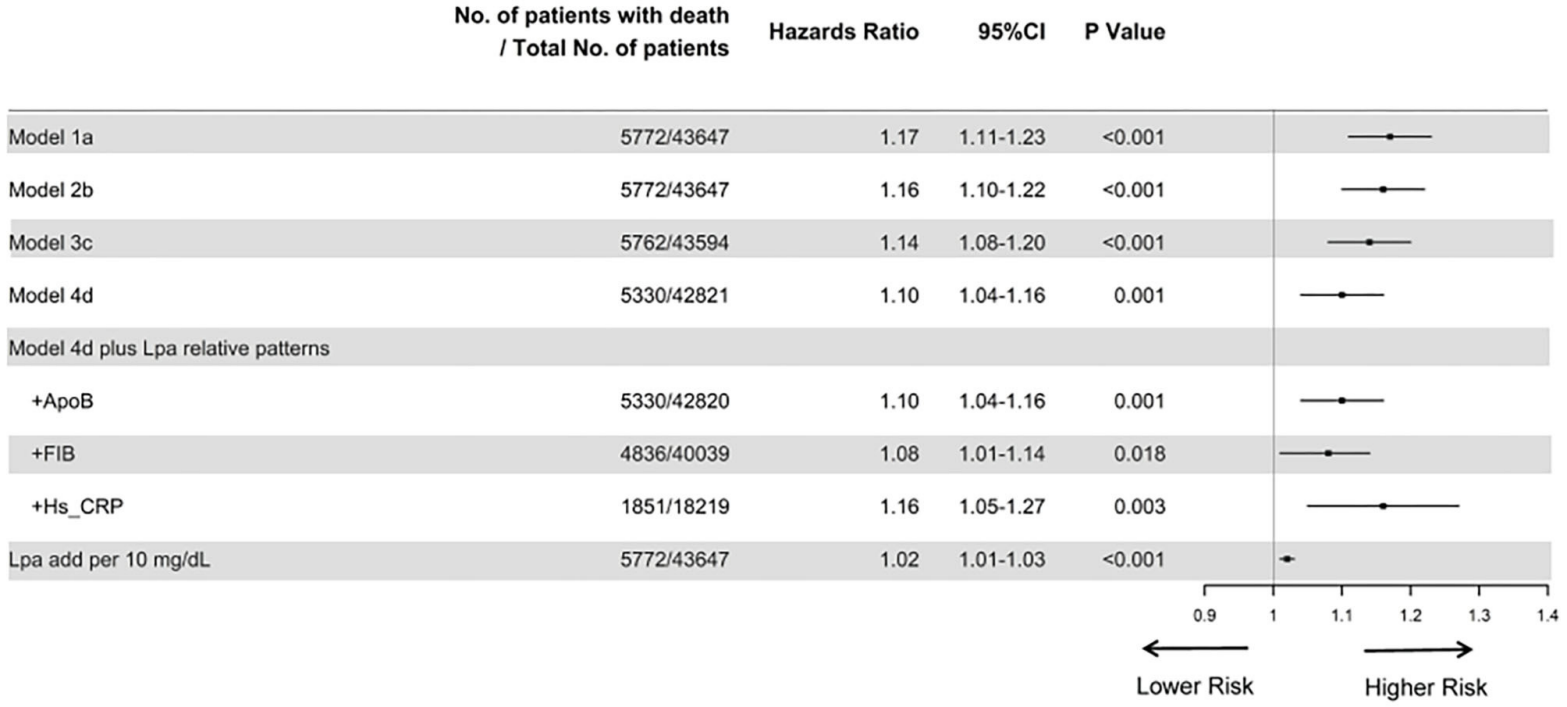
Cox proportional hazard ratios for long-term all-cause mortality in different models. Model 1 was unadjusted; model 2 was only adjusted for age and gender; Model 3 was adjusted for age, gender, total cholesterol, triglycerides, apolipoprotein A and low-density lipoprotein cholesterol; and model 4 was adjusted for age, gender, hypertension, acute myocardial infraction, diabetes mellitus, percutaneous coronary intervention, hemoglobin, white blood cell, congestive heart failure, total cholesterol, triglyceride, apolipoprotein A, low-density lipoprotein cholesterol, high-density lipoprotein cholesterol, chronic kidney disease, angiotensin-converting enzyme inhibitor/angiotensin receptor blockers, β-blockers, and statins. Furthermore, we additionally adjusted apolipoprotein B, fibrinogen, and hypersensitive C-reactive protein, respectively, based on model 4. Finally, we unadjusted for lipoprotein(a) additionally per 10 mg/dL.

The prevalence of total mortality in the lower and higher lipoprotein(a) groups according to the median of lipoprotein(a) levels were 6.4 and 9.0%, respectively. As determined by Kaplan–Meier analysis, the elevated lipoprotein(a) levels (≥15 mg/dL) led to worse long-term prognosis (log-rank *P* < 0.001, Figure 1). The univariate regression analysis showed that 25 variables (including age, sex, etc.) were significantly associated with the primary endpoint (Supplementary Table 1). In the analyses of univariate and multivariate models, we observed an approximate linear association between lipoprotein(a) levels and long-term all-cause mortality (Figure 2). According to the results of the multivariable-adjusted models, lipoprotein(a) levels ≥15 mg/dL increased the risk of all-cause death by 10% compared with lipoprotein(a) levels <15 mg/dL (aHR, 1.10; 95%CI, 1.04–1.16) (Figure 3).

**Figure 4:**
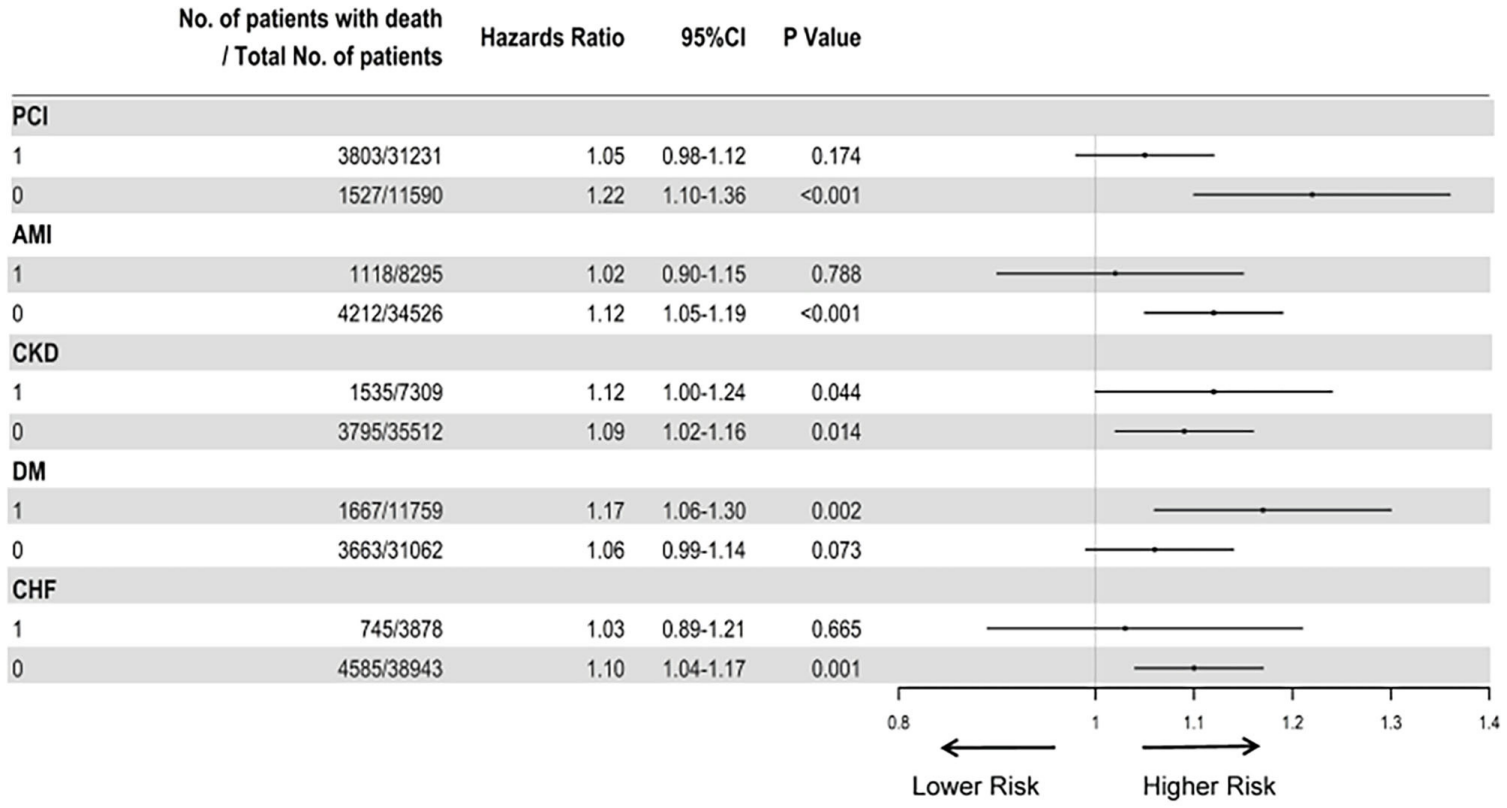
Cox proportional hazard ratios for long-term all-cause mortality in different subgroups. The analysis in different subgroups was adjusted for age, gender, hypertension, acute myocardial infraction, diabetes mellitus, percutaneous coronary intervention, hemoglobin, white blood cell, congestive heart failure, total cholesterol, triglyceride, apolipoprotein A, low-density lipoprotein cholesterol, high-density lipoprotein cholesterol, chronic kidney disease, angiotensin-converting enzyme inhibitor/angiotensin receptor blockers, β-blockers, and statins.

The subgroup analyses also suggested that the results did not change substantially among patients who underwent PCI (aHR, 1.95; 95%CI, 0.98–1.12), AMI (aHR, 1.02; 95%CI, 0.90–1.15), non-DM (aHR, 1.06; 95%CI, 0.99–1.14), and CHF (aHR, 1.10, 95%CI, 1.04–1.17) (Figure 4).

## Discussion

To our knowledge, the present study provides the first systematical evaluation of the association of moderately elevated baseline plasma lipoprotein(a) and all-cause mortality in a large cohort of CAD patients. Our data showed that lipoprotein(a) levels ≥15 mg/dL were strongly associated with all-cause death in patients with CAD. Similar results were observed in the subgroup analysis [non-PCI, CKD (yes or no), non-AMI or DM and non-CHF).

An earlier study indicated that lipoprotein(a) is a residual risk factor in patients who achieved target lipid levels by the time of treatment by PCI (17). Additionally, another previous study showed that lipoprotein(a) was significantly associated with long-term adverse cardiovascular outcomes among CAD patients who received statin therapy after PCI (18). Recent dyslipidemia relative guidelines indicated that lipoprotein(a) levels were an optional risk stratification of patients with high atherosclerotic cardiovascular disease risk, and when the lipoprotein(a) concentration is above 50 mg/dL, it would be regarded as a significant risk factor of prognosis (8). Because previous studies mainly included the general population or limited samples of CAD patients, the associations between specific elevated plasma levels of lipoprotein(a) and long-term all-cause mortality in CAD are limited (5, 7, 19–21). In addition, there is no definitive clinical recognition of the cutoff value of lipoprotein(a) associated with long-term all-cause mortality. Therefore, we included large samples (more than 40,000) of CAD patients to further identify the relationship between lipoprotein(a) levels and long-term prognosis. After adjusting for demographic characteristics, medical history, laboratory tests, and medication variables (8, 22), the moderately elevated baseline lipoprotein(a) levels (≥15 mg/dL) remained significantly associated with an unfavorable prognosis. Furthermore, there was good statistical evidence for a similar result in different subgroups [non-PCI, CKD (yes or no), non-AMI or DM and non-CHF].

Our study found that lipoprotein(a) is an independent risk factor for all-cause mortality, which should be tested among CAD patients. Furthermore, CAD patients with lipoprotein(a) level ≥15 mg/dL had a higher risk of all-cause mortality, and they might need a lipoprotein(a)-lowering treatment. Many studies have revealed that PCSK9i can decrease the lipoprotein(a) concentration, which may considered for practice in CAD patients with moderately elevated lipoprotein(a) (23, 24).

Lipoprotein(a) is a plasma lipoprotein composed of apolipoprotein(a) covalently bound to apolipoprotein B-100, a low-density lipoprotein-like particle. Previous studies have shown a significant relationship between high lipoprotein(a) levels and cardiovascular disease (4, 25). Patients with high levels of lipoprotein(a) tend to have more severe cardiovascular conditions, which may lead to an increased risk of all-cause death. It is generally believed that lipoprotein(a) participates in the pathophysiological process of coronary artery disease by promoting atherosclerosis or inflammation (26). In addition, lipoprotein(a) may promote the thrombotic state through a variety of mechanisms, including inhibition of the fibrinolytic system and enhancement of tissue factor-mediated pathways (27, 28). Although high lipoprotein(a) levels may protect against bleeding events, thrombosis plays a more crucial role in the poor prognosis among patients with existing CAD.

### Strength and Limitation

This study examined for the first time the association of baseline plasma lipoprotein(a) and all-cause mortality in a large cohort of CAD patients. The abundant data extracted from medical records allowed us to control for a variety of confounders in the analyses. There are several limitations to this study. First, information about cause-specific death was not available in this study, which restricted our ability to examine the association of lipoprotein(a) with cause-specific death, such as CVD mortality. Second, only in-hospital baseline lipoprotein(a) was contained in our study. Therefore, we could not know the status of lipoprotein(a) after discharge and the effects of its change. However, we are further collecting the follow-up lipoprotein(a) information after discharge. Third, although we have adjusted for many confounders in the analyses, residual confounding due to unmeasured factors was still available (such as socioeconomic factors, body mass index, and lifestyle factors).

## Conclusion

Moderately elevated plasma lipoprotein(a) levels were significantly associated with an increased risk of all-cause mortality among patients with confirmed CAD, and moderately elevated baseline plasma lipoprotein(a) (≥15 mg/dL) could identify the risk of long-term all-cause mortality in patients with CAD. Our finding highlighted the significance of plasma lipoprotein(a) as an independent and long-term prognostic indicator for CAD patients. Future studies are needed to confirm our findings.

## Data Availability Statement

The raw data supporting the conclusions of this article will be made available by the authors, without undue reservation.

## Ethics Statement

Ethical review and approval was not required for the study on human participants in accordance with the local legislation and institutional requirements. The patients/participants provided their written informed consent to participate in this study.

## Author Contributions

YL, JLiu, NT, and JC conceptualized the research idea and study design. JLiu, ZL, LL, JLia, SC, BW, QL, HH, and ZH contributed to data acquisition. JLiu and YL contributed to data analysis/interpretation. DX performed the statistical analysis. YL, JC, and NT took charge of supervision and mentorship. XY, GAT, and BL provided writing guidance. Each author contributed important intellectual content during manuscript drafting or revision and accepts accountability for the overall work by ensuring that questions pertaining to the accuracy or integrity of any portion of the work are appropriately investigated and resolved.

## Conflict of Interest

The authors declare that the research was conducted in the absence of any commercial or financial relationships that could be construed as a potential conflict of interest.

## Publisher's Note

All claims expressed in this article are solely those of the authors and do not necessarily represent those of their affiliated organizations, or those of the publisher, the editors and the reviewers. Any product that may be evaluated in this article, or claim that may be made by its manufacturer, is not guaranteed or endorsed by the publisher.
