# Supplementary material for: Coronary Artery Disease: Optimal Lipoprotein(a) for Survival—Lower Is Better? A Large Cohort With 43,647 Patients
Source: Front Cardiovasc Med. 2021 Aug 31;8:670859. doi: 10.3389/fcvm.2021.670859 (PMC8438333; doi:10.3389/fcvm.2021.670859)

**Supplemental Table S1.** Univariate cox regression analysis of long-term all-cause mortality

| Variables | Univariate | |
| --- | --- | --- |
|  | HR（95% CI） | P value |
| Age | 1.03(1.03- 1.03) | ＜0.001 |
| Female | 0.94(0.89- 1.00) | 0.063 |
| AMI | 1.25(1.17- 1.32) | ＜0.001 |
| CHF | 2.59(2.41- 2.78) | ＜0.001 |
| Hypertension | 1.19(1.13- 1.25) | ＜0.001 |
| DM | 1.30(1.23- 1.37) | ＜0.001 |
| PCI | 0.88(0.84- 0.94) | <0.001 |
| CKD | 2.19(2.07- 2.31) | ＜0.001 |
| lipoprotein(a) | 1.00(1.00- 1.00) | ＜0.001 |
| WBC | 1.05(1.04- 1.06) | ＜0.001 |
| HGB | 0.98(0.98-0.98) | ＜0.001 |
| CHOL | 0.95(0.93-0.97) | ＜0.001 |
| TRIG | 0.920.90-0.95) | ＜0.001 |
| APOA | 0.66(0.6-0.73) | ＜0.001 |
| APOB | 0.87(0.78-0.98) | 0.018 |
| LDLC | 0.96(0.93-0.99) | 0.002 |
| HDLC | 0.83(0.75-0.91) | ＜0.001 |
| HbA1c | 1.08(1.06-1.11) | ＜0.001 |
| URIC | 1.00(1.00-1.00) | ＜0.001 |
| eGFR | 0.98(0.98-0.98) | ＜0.001 |
| ACEI/ARB | 0.87(0.82-0.91) | ＜0.001 |
| Beta-blockers | 0.85(0.80-0.91) | ＜0.001 |
| Statin | 0.73(0.66-0.81) | ＜0.001 |

Abbreviation: AMI, acute myocardial infarction; CHF, congestive heart failure; DM, diabetes mellitus; PCI, percutaneous coronary intervention; CKD, chronic kidney disease; WBC, white blood cell; HGB, hemoglobin; CHOL, serum total cholesterol; TG, Triglyceride; APOA, apolipoprotein A; APOB, apolipoprotein B; LDL-C, low-density lipoprotein Cholesterol; HDL-C, hight-density lipoprotein cholesterol; eGFR, estimated glomerular filtration rate; ACEI/ARB, angiotensin-converting enzyme inhibitor/angiotensin receptor blocker.

**Supplemental Table S2.** The ICD-10 codes information of diagnoses.

| Diagnosis | ICD-10 codes |
| --- | --- |
| Hypertension | I10.x00, I10.x05, I10.x04, I10.x03, I11.900, I10. 13, I12.900, H35.004, I12.903, I15.900, I67.400, I13.900, I15.800, I11.000, I12.000, I10.x06, I10.x01, I13.100, I10.x04, I10. 03, I10.x05, I10.x03, R03.000, I11.901, I10.x09, I10.x07, I10.x00, I15.102, I12.900, I11.900, I11.002, I12.000, I10.x11, I13.900, I15.103, I15.200, I11.000, I11.001, I10.x12, I10.x10, I13.000, |
| Diabete mellitus | E11.900, E14.900, E11.901, E11.300, E11.301+H36.0, E11.401+G63.2, E11.501+I79.2, E11.601, E11.700, E11.200+N08.3, E10.900, E13.905, E11.800, E13.903, E11.400, E11.200+N08.3, E13.300+H36.0, E13.200+N08.3, E11.100, E13.907, E11.10001, E11.60001, E14.10001, E11.500, E10.401+G63.2, E11.403+G63.2, E13.400+G63.2, E13.500, E13.700, Z83.300, E11.502, E11.60002, E10.201, E11.901, N08.301*, E11.900, E11.700, E11.200, E11.90002, E11.201+N08.3, E14.900, E13.900, E14.300, E14.800, E13.200, E11.800, O24.300, E14.200, O24.100, E13.800, E11.503, E11.400, E13.600, E14.600, E11.302+H28.0, E14.500, E14.400, E11.500, E11.101, E13.201+N08.3, E11.70001, E14.700, E11.502+I79.2, E13.400, E11.100, E11.103, E11.300, E13.300, E10.400, E13.500, E11.505, E12.000, E11.600, E13.700, E11.604, E11.402+G99.0, R73.003 |

**Supplemental Figure S1.** Flow of participants through the trial.


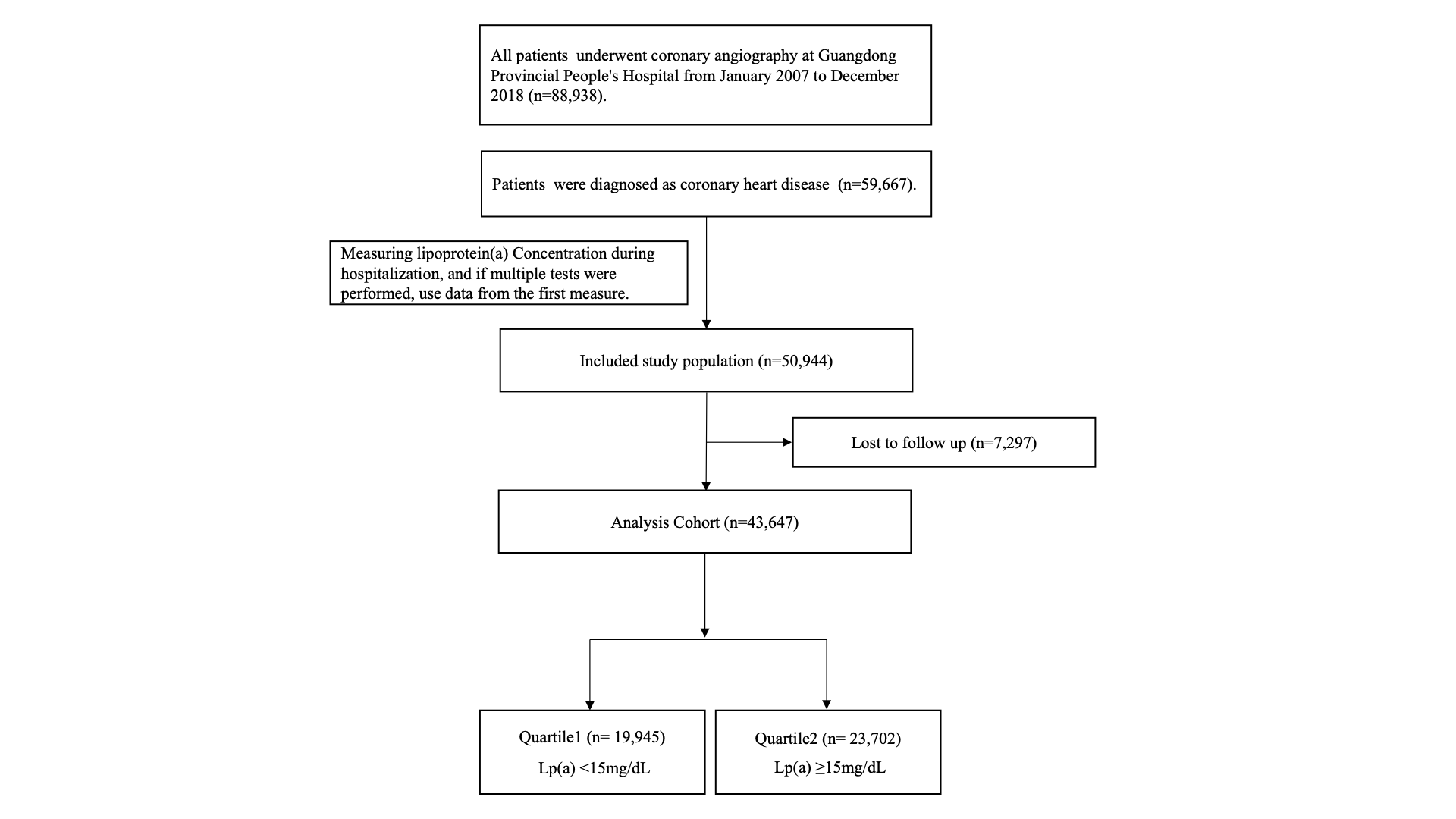


**Supplemental Figure S2.** The pyramid of plasma levels of lipoprotein(a)


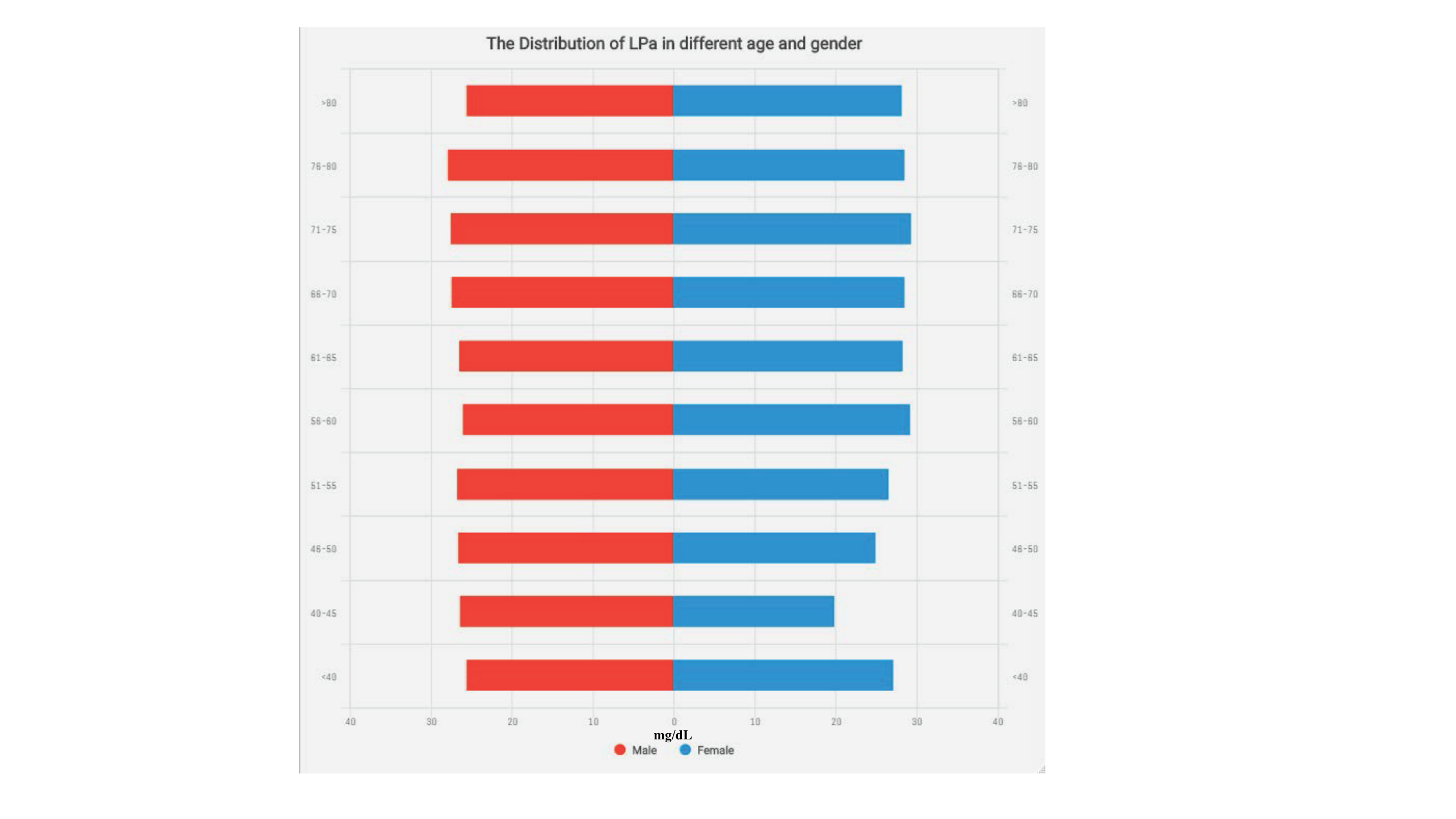

Supplement: Supplementary file 1 [file Data_Sheet_1.docx]
